# Of Diseases, Supposed to Be Venereal, Produced by Transplanted Teeth

**Published:** 1786

**Authors:** John Hunter


XVI. *Of Diseases, supposed to be venereal, produced by transplanted Teeth.* Vide *A Treatise on the Venereal Disease, by* John Hunter. 4to. *London*, 1786.

SINCE the practice of transplanting teeth has been adopted in this country, several cases have occurred in which the venereal infection has been supposed to be communicated in this way, and they have been treated accordingly; but the ingenious and respectable

 author author of the work before us is of opinion, that, when all the circumſtances attending them are conſidered, there will be found, in all of them, ſomething which is not exactly ſimilar to the uſual appearance of the venereal diſeaſe when received in the common way.

In all the caſes of this kind which have fallen under Mr. Hunter's obſervation, the time of local affection, after the inſertion of the tooth, has been almoſt uniformly a month, which he conſiders as too long a period for the venereal infection to take effect in at a medium. He obſerves farther, that where conſtitutional ſymptoms have been produced in theſe caſes, theſe alſo have followed the local ſymptoms more cloſely, and with more regularity as to time, than could have been expected if they had been venereal.

The firſt caſe of this kind which came under his care was that of a lady, who had one of the bicuſpidati tranſplanted. The tranſplanted tooth, we are told, faſtened very well. About a month after ſhe caught cold after dancing, and had a fever, which laſted near ſix weeks. In this time ulceration in the gum and jaw took place, though it was not then known; but when ſhe was beginning to recover it was obſerved, obferved, that not only the gum and focket of this tooth were difeafed, but alfo thofe of the tooth next to it. The two teeth were taken out, and the fockets of both afterwards exfoliated, but the parts were very backward in healing. This circumftance gave rife to various opinions, the principal of which was, that the complaint was venereal. In the mean time, a rifing of the indolent node kind appeared on one of her legs, and was confidered as a corroborating proof that the cafe was venereal: but Mr. Hunter being of a different opinion, recommended fea bathing, which was accordingly had recourfe to, and the patient, we are told, got perfectly well, and has remained fo ever fince.

The fecond inftance he met with of this difeafe was alfo in a young lady. In this, as in the former cafe, the tranfplanted tooth faftened extremely well; but, at the end of about a month, the gum began to ulcerate, leaving the tooth and focket bare. The ulcer continued, and blotches appeared on the fkin, as did alfo ulcers in the throat. The difeafe was treated as venereal, and the patient at length got well, but not, it feems, till after the fymptoms had recurred feveral times. This return of the

ſymptoms in this patient, after continued courſes of mercury, was much more frequent, the author obſerves, than is uſual in venereal caſes; and he had his ſuſpicions, he tells us, all along that the complaint was ſcrophulous.

In the third caſe, which was that of a gentleman, the tranſplanted tooth remained, without giving the leaſt diſturbance, for about a month, and then the edge of the gum began to ulcerate, and the ulceration continued till the tooth dropped out. Some time after ſpots appeared almoſt every where on the ſkin; they had not, however, it ſeems, the true venereal appearance, but were redder, or more tranſparent, and more circumſcribed. He had alſo, we are told, a tendency to a hectic fever, and complained of reſtleſſneſs, want of ſleep, loſs of appetite, and head-ach. After he had tried different remedies without finding relief, he was put under a courſe of mercury, and his complaints diſappearing, he was thought to be well; but ſome time after the ſame appearances returned as at firſt, with the addition of ſwelling in the bones of the metacarpus. He was then put under another courſe of mercury, more ſevere than the former, and in the uſual time all the ſymptoms again diſappeared. Several months months after the ſame eruptions came out again, but not in ſo great a degree as before, and without any other attendant ſymptoms. He now had recourſe again to mercury for the third time; but he took only ten grains of corroſive ſublimate in the whole, and got quite well. The time between his firſt taking mercury, and his being cured, was, we are told, a ſpace of three years.

Could this caſe, aſks the author, be venereal? — The circumſtance of the two firſt courſes of mercury having removed the eruptions, would, he obſerves, ſeem to prove that it was; but the third courſe, which conſiſted of only ten grains of corroſive ſublimate, having alſo removed them, ſeems, he thinks, to prove that it could not be venereal; for, if it had, the appearances which returned after the ſecond courſe, in which a conſiderable quantity of mercury had been given, would, he is of opinion, not have yielded to ten grains of corroſive ſublimate.

The ſubject of the fourth caſe mentioned by the author was a young lady, whoſe gum began to ulcerate at about the ſame diſtance of time after the tranſplanting of a tooth as in the preceding caſes. The ſurgeon who was firſt conſulted

ſulted adviſed the uſe of mercurial medicines; but Mr. Hunter, who was afterwards deſired to ſee her, adviſed that mercury ſhould not be had recourſe to, and this he did to aſcertain the nature of the caſe; for if ſhe took mercury, and got well, it would, he obſerved, be adding one more to the number of the ſuppoſed venereal caſes that had ariſen from ſuch a cauſe. He recommended the extraction of the tooth, that it might be ſeen what effects would be produced by the removal of the firſt cauſe. The tooth was accordingly drawn, and the gum healed as faſt as any common ulcer, and has remained well ever ſince.

With reſpect to this caſe, Mr. Hunter remarks, that if the patient had gone through a courſe of mercury, ſhe would probably alſo have got well; for the tooth, in the time neceſſary for completing the courſe of mercury, would have dropped out; and if this had really happened, he thinks, we need not heſitate to affirm, that the caſe would have been conſidered as venereal.

In the fifth caſe, which was that of a young lady, eighteen years of age, who had one of the inciſores tranſplanted, ulceration of the gum took place ſix or ſeven weeks after the operation, operation. The tooth was immediately extracted, the bark was given without any other medicine, and ſhe got well in a few weeks.

The ſixth caſe was that of a gentleman, aged twenty-three, who had the two front inciſores tranſplanted, and within about the ſame ſpace of time after the operation, as in the former caſes, had an ulceration of the gum, the edges of which ſloughed off. A ſurgeon who was conſulted recommended the bark, and the patient, without taking any other medicine, got well, we are told, in nearly the ſame time as the ſubjects of the fourth and fifth caſes, who had the teeth taken out. The gums, in this caſe, it is added, healed perfectly, but were conſiderably ſhorter.

The ſeventh and laſt caſe mentioned by the author is that of a young lady, whom he ſaw juſt as the edge of the gum was beginning to ulcerate. He recommended the frequent application of a ſolution of corroſive ſublimate to the ulceration; but as this did not ſtop its progreſs, ſhe applied, he tells us, to Dr. Watſon, from whoſe account of the caſe, publiſhed in the Medical Tranſactions *, he takes his ma-

* See the next article.

terials terials to argue upon; and his remarks on it tend to prove that this cafe was not venereal.

In the firft place, Mr. Hunter contends that the progrefs of the ulceration in the mouth, which was the firft fymptom, was by much too rapid for a venereal ulcer; for it muft be confidered, he obferves, if venereal, fimply as a chancre or local affection.

In the next place, he is of opinion that the conftitutional affections followed the local by much too foon to allow us to fuppofe them to have been venereal; and farther, he obferves, that the appearances from the conftitution, when they did take place, were much more violent and rapid in their progrefs than any fimilar appearances he has ever feen in venereal cafes.

With refpect to the cure, Mr. Hunter remarks, that the quantity of mercury employed would be inadequate to the cure either of chancres on the penis, or of venereal fores on the fkin, making fo rapid a progrefs as the ulcers in the mouth and the fores on the fkin did in this cafe. And if we take into confideration the effect produced upon the patient's health, with the termination of the whole, he thinks we fhould pronounce the cafe not venereal; for for the ſpecific circumſtances, if it was venereal, were, he contends, juſt as uncommon as the mode of catching it.

After having conſidered the caſes of thoſe who had the teeth tranſplanted, Mr. Hunter proceeds to conſider the perſons from whom the teeth were taken; and his remarks here tend to throw additional light on the ſubject. "Let me ſuppoſe," ſays he, "that the young "girls from whom the teeth were taken really "had the lues venerea, and that the teeth "were of courſe alſo infected." This is a ſuppoſition, he obſerves, moſt unfavourable to his real opinion; but he thinks that even if this had actually been the caſe, there could have been no difference between the gums of the perſons from whom the teeth were taken, and the gums of thoſe who received them. If the ulceration, he aſks, took place in the latter from contamination, would not the ſockets of the perſons from whom the teeth were taken likewiſe have ulcerated? But this, we are aſſured, did not happen in any of the caſes.

Mr. Hunter has here ſuppoſed the teeth capable of being contaminated with the lues venerea, although he believes they have never yet been ſeen to have this diſeaſe primarily, but only in confequence of its breaking out fomewhere elfe, as in the mouth, throat, or nofe, and from thence fpreading to them; "but ftill," fays he, "if they are capable of "having the difeafe, and communicating it to "others, it becomes very extraordinary that "thefe people fhould have hit upon the only "fuch teeth that probably were ever fo conta-"minated;" and he thinks it ftrange that the difeafe fhould break out in the receivers, and not in the givers. It is alfo fingular, he adds, that an ambiguity fhould follow this difeafe in all its ftages; in the mode of its being received, in its appearance, and in its cure.

After having thus fully confidered the difeafe in queftion, Mr. Hunter fums up all the arguments in favour of its not being venereal.

Firft, he obferves, that two of the patients, whofe cafes were fimilar to the others in their origin, recovered without any medicine; and fecondly, that they who feemed to be cured by mercury had not a treatment exactly fimilar to what is followed in cafes where the difeafe is indifputably the lues venerea: thirdly, he confiders it as impoffible for parts to have the power of contaminating, which have not themfelves affumed what he terms the difeafed action; tion; and laftly, he obferves, that the parts contaminating were never known to be contaminated themfelves.

But it muft be nearly the fame thing, Mr. Hunter obferves, to thofe who want to have teeth tranfplanted, whether his reafoning on this fubject is juft or not; for a difeafe in confequence of the operation moft certainly has taken place; and in fome cafes this has been worfe, or cured with more difficulty, than the lues venerea ufually is: and whatever the difeafe may be, he as yet knows of no mode of prevention, except the drawing of the tooth early, and that has been tried, he fays, in one cafe only, and in that cafe was fuccefsful.

" From this account," fays Mr. Hunter, " many may be deterred from having this ope-" ration performed; in that light no evil can " arife, except being mortified that no means " of relief can be had recourfe to in cafes of " bad teeth; but it is to be remembered, that " this is a publication of all the unfuccefsful " cafes, which is the very reverfe of what is " generally practifed in medical books; and " they are mentioned upon no other principle " than that the difeafe, when it happens, may " not be improperly managed."

It may be asked what is this disease? This is a question, Mr. Hunter observes, which it may be more difficult to answer than what it is not. He would say, however, "that a sound tooth "transplanted may occasion such an irritation "as shall produce a species of disease, which "may be followed by the local complaints "above mentioned."

